# P-617. Multi-Parametric Diagnostic Algorithm for Early Detection of Severe Acute Respiratory Infections in Children: A Prospective Multi-Center Study in Resource-Limited Settings

**DOI:** 10.1093/ofid/ofaf695.830

**Published:** 2026-01-11

**Authors:** Barnali Mitra, Debdeep Mitra

**Affiliations:** Command Hospital Western command Chandimandir, Chandigarh, Chandigarh, India; Command Hospital Air Force Bangalore, Bangalore, Karnataka, India

## Abstract

**Background:**

Pediatric respiratory infections remain a leading cause of morbidity and mortality in resource-limited settings, with diagnostic challenges due to limited laboratory infrastructure and delayed access to healthcare. This study evaluates a novel multi-parametric diagnostic algorithm combining clinical indicators with point-of-care biomarkers for early identification of severe acute respiratory infections (SARIs) in children under 5 years.

**Methods:**

We conducted a prospective, multi-center observational study across 12 healthcare facilities (8 rural, 4 urban) in five states of India from June 23 to February 25. A total of 3,240 children aged 3-59 months presenting with respiratory symptoms were enrolled. Our diagnostic algorithm integrated clinical parameters (respiratory rate, oxygen saturation, chest indrawing) with point-of-care biomarkers (CRP, PCT, and neutrophil-lymphocyte ratio) in a mobile application-based scoring system. The algorithm's performance was compared against a reference standard comprising radiological findings, comprehensive laboratory testing, and 14-day clinical outcomes.

**Results:**

The multi-parametric algorithm demonstrated 92.4% sensitivity and 89.7% specificity for identifying severe respiratory infections requiring intervention, significantly outperforming standard clinical assessment alone (sensitivity 78.6%, specificity 72.3%; p< 0.001). Implementation of the algorithm reduced time to appropriate treatment initiation from 8.4 to 2.7 hours (p< 0.001) and decreased unnecessary antibiotic use by 41.6%. The algorithm effectively distinguished bacterial from viral etiologies with 87.3% accuracy. Cost analysis revealed a 38.2% reduction in overall healthcare expenditure per case. Field health workers demonstrated 96.8% proficiency in algorithm application after a standardized 4-hour training program.

**Conclusion:**

Our novel multi-parametric diagnostic algorithm provides an accurate, cost-effective approach for early identification of severe pediatric respiratory infections in resource-limited settings. The integration of clinical parameters with accessible point-of-care biomarkers enables rapid triage and appropriate management decisions where comprehensive laboratory facilities are unavailable.

**Disclosures:**

All Authors: No reported disclosures

